# #CardioOncology: Twitter chat as a mechanism for increasing awareness of heart health for cancer patients

**DOI:** 10.1186/s40959-020-00072-w

**Published:** 2020-09-09

**Authors:** Claire C. Conley, Neha G. Goyal, Sherry-Ann Brown

**Affiliations:** 1 Department of Oncology, Georgetown University, Washington, DC USA; 2 Helen Diller Family Comprehensive Cancer Center, University of California San Francisco, San Francisco, CA USA; 3 Cardio-Oncology Program, Division of Cardiovascular Medicine, Medical College of Wisconsin, 8701 W Watertown Plank Road, Wauwatosa, WI 53226 USA

**Keywords:** Cardio-oncology, Cardiology, Cancer, Cancer survivorship, Social media, Twitter

## Abstract

Consideration of heart health for cancer survivors is increasingly important, as improved cancer survivorship has resulted in a growing number of survivors affected by cardiovascular disease. However, there is limited knowledge of cardio-oncology among both patients and a variety of health professionals. Thus, efforts are needed to increase awareness about cardio-oncology. Social media represents one potential opportunity to disseminate information about cardio-oncology to a large audience. We highlight one example of a social media educational/advocacy campaign conducted on Twitter (a “Twitter Chat”) that garnered nearly 1.2 million impressions (views by Twitter users) in just 24 h. We provide both quantitative and qualitative data to support the efficacy of using Twitter for such educational/advocacy campaigns, and describe key features that contributed to its success. Twitter Chats inexpensively utilize innovative technology to provide education and foster community. Long-term studies are needed to understand whether Twitter Chats can change knowledge and behavior related to cardio-oncology.

Cardio-oncology is an emerging subspecialty focusing on the prevention and management of cardiovascular injury from cancer therapies [1, 2]. It is increasingly important to consider cancer survivors’ heart health, as improved cancer survivorship has resulted in a growing number of survivors affected by cardiovascular disease [3]. In addition, cardiovascular disease is the leading cause of mortality in cancer survivors [4]. To address this phenomenon, dedicated cardio-oncology programs are rapidly emerging to treat cancer patients with de novo and preexisting cardiovascular disease [5, 6]. Such programs are interdisciplinary and often include cardiologists, hematologists/oncologists, nurses, dietitians, pharmacists, and social workers. Together, these experts can address both prevention and treatment of cancer therapy-related cardiotoxicity. However, there is room for improvement in cardio-oncology knowledge among both patients [7, 8] and health professionals [9, 10]. Thus, efforts are needed to increase awareness about the intersection of heart health and cancer.

Social media represents one potential opportunity to disseminate medical information to a large audience. Social media offers a way to distinguish and disseminate medical information much more rapidly and broadly than through traditional peer review and subsequent health communication efforts [11]. In 2019, 72% of U.S. adults used at least one social media site [12]. Young adults were among the earliest social media adopters and continue to use these sites at high levels (90% of persons age 18–29); usage by older adults has also increased in recent years (40% of persons age 65+) [12]. Social media use is also fairly equivalent across racial/ethnic groups (73% of White adults, 70% of Hispanic adults, and 69% of Black adults) and income strata (ranging from 68% for those with income <$30,0000/year to 83% for those with income $50,000–$74,999/year) [12].

The microblogging website Twitter (http://twitter.com) has been embraced by interdisciplinary professionals in cardio-oncology around the world. Twitter provides opportunities for networking, support, collaboration, and education for individuals across specialties, including cardiology, hematology, and medical and radiation oncology. Practitioners from these specialties have pioneered the use of the hashtags^1^ #CardioOnc, #CardioOncology, and others representative of the multidisciplinary field to provide a framework for ongoing discussions [13]. Thus, Twitter has brought together the global cardio-oncology community and has helped increase awareness about the emerging subspecialty, even during the COVID-19 pandemic [14].

In addition to posts by individual users, Twitter offers a unique opportunity to disseminate information in real time through the “Twitter Chat” format. Twitter Chats are live, public discussions that take place on Twitter at a predetermined time, about a predetermined topic. In essence, Twitter Chats are panel discussions that have been moved into the digital world of Twitter. All posts during the Twitter Chat include a designated hashtag that enables participants to follow along with the conversation. Over the course of a Twitter Chat, a moderator typically posts several questions. Participants concurrently respond to each of the questions and interact with others’ responses for a dynamic conversation about the topic. While open to the public, Twitter Chats often utilize featured guests to facilitate the conversation and ensure well educated and informed engagement.

In February 2020, the Society of Behavioral Medicine (SBM) hosted a Twitter Chat focusing on cardio-oncology. SBM began hosting Twitter Chats in May 2018, utilizing the hashtag #BehavioralMedChat. Since then, there have been 20 #BehavioralMedChat events with over 8000 unique tweets. Cardio-oncology was chosen as the #BehavioralMedChat topic for February 2020, due to increasing interest in the topic within SBM. Given the interdisciplinary nature of both SBM and cardio-oncology, this Twitter Chat was designed to appeal to a diverse audience, including researchers, clinicians, cancer patients and survivors, and the lay public. As such, we invited six guests including two cardio-oncologists (Doctors of Medicine [MDs]), a registered dietitian (RD), a registered social worker (RSW), and two patient advocates. The four discussion questions for the chat were similarly developed with a diverse audience in mind, and were designed to be answerable by both experts and non-experts (Fig. 1).

**Fig. 1:**
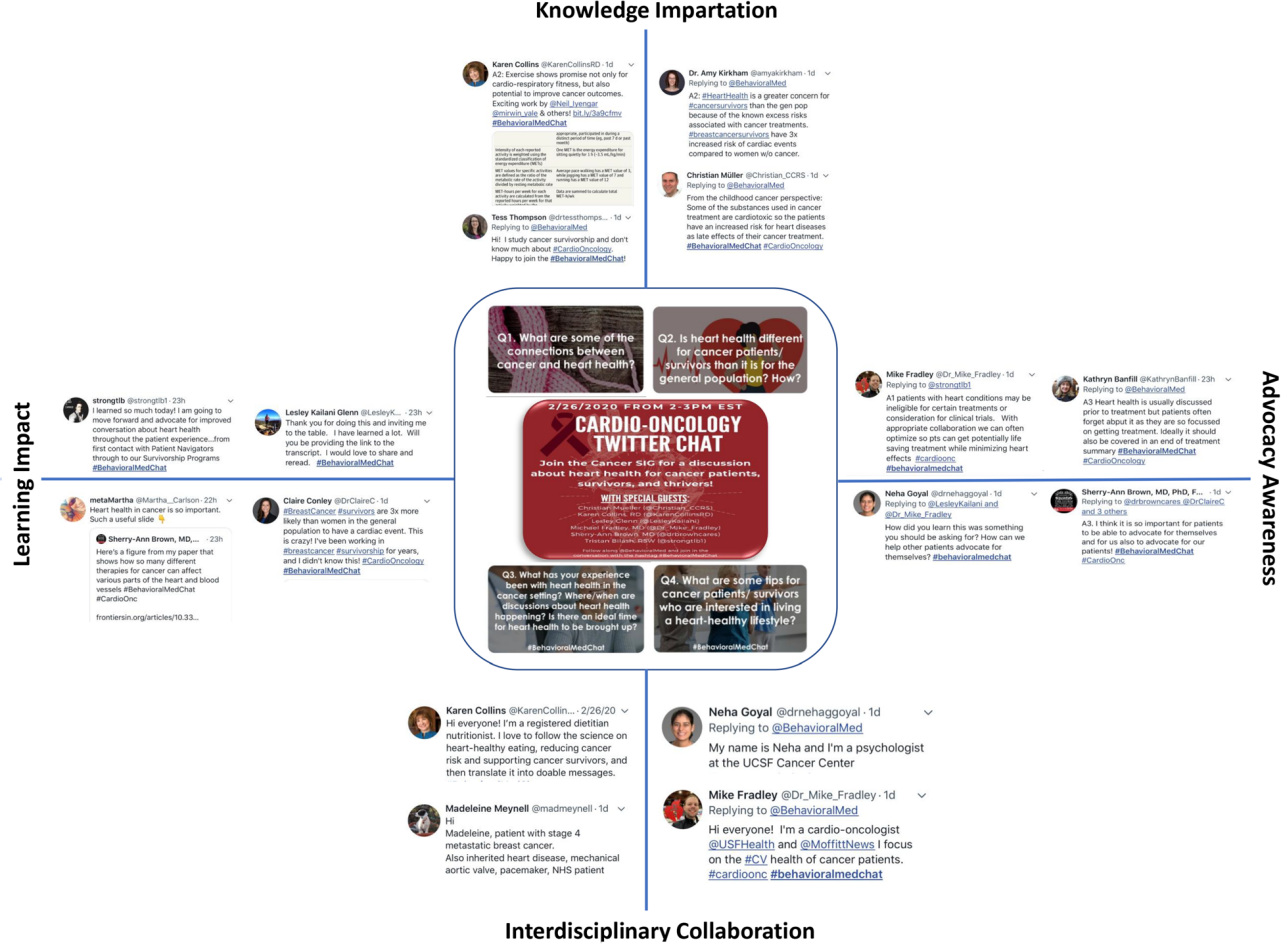
KAIL. Representative responses to each of the four questions posted in the February 2020 Society of Behavioral Medicine (SBM) monthly Twitter Chat (#BehavioralMedChat), which focused on cardio-oncology. Four themed domains or categories emerged: knowledge impartation (K), advocacy awareness (A), interdisciplinary collaboration (I), and learning impact (L), termed KAIL

We collected data on the cardio-oncology #BehavioralMedChat from the Symplur Healthcare Hashtag Project [15]. Symplur tracks and archives tweets associated with registered health care-related hashtags. Symplur has a free and publicly available interface, where anyone can register and search for health care-related hashtags. To access hashtag-specific Twitter analytics, Symplur can be queried for defined time frames. We queried Symplur for data on the #BehavioralMedChat hashtag from February 26, 2020 at 12:00 pm ET (the start of the cardio-oncology chat) through February 27, 2020 at 12:00 pm ET. In the 24-h period including the 1-h cardio-oncology Twitter Chat, 105 unique participants tweeted 603 times, for an average of 6 tweets per participant. Twitter provides a statistic called “impressions” which specifies the number of times that a tweet was seen by a Twitter user. The cardio-oncology Twitter Chat had 1.175 million impressions in 24 h.^2^ For comparison, a recent (February 12, 2020) Twitter Chat on heart failure (#HFChat2020) sponsored by the Heart Failure Society of America and the American Association of Heart Failure Nurses generated 1.429 million impressions in 24 h. Thus, #HFChat2020 had 1.2 times the number of impressions generated by our cardio-oncology Twitter Chat. This difference may be due to heart failure being a more longstanding subspecialty within Cardiology than the relatively new Cardio-Oncology subspecialty. In addition, the heart failure Twitter Chat featured the participation of several large organizations with substantial presence on Twitter, such as the American College of Cardiology’s patient education initiative CardioSmart (@CardioSmart) and Boehringer Ingelheim (@boehringerus; a pharmaceutical company).

The cardio-oncology Twitter Chat can also be compared to prior SBM Twitter Chats. The number of impressions generated by the three prior SBM Twitter Chats were 482,206 (February 18, 2020), 621,562 (December 9, 2019), and 308,945 (December 6, 2019). Thus, the cardio-oncology Twitter Chat generated nearly 2.5 times more impressions than the average of the three prior SBM Twitter Chats. This high number of impressions in a short amount of time is likely attributable to the cardio-oncology Twitter Chat’s interdisciplinary audience with a high number of non-overlapping followers engaging with intentionally high-quality material.

Another important Twitter analytic is “engagement rate”, or the number of times that a Tweet was engaged with (i.e., clicks, likes, replies, retweets, etc.) divided by the number of impressions. Thus, engagement rate demonstrates the value of posted content through followers’ interactions with that content. The engagement rate for posts was obtained in one of two ways. The engagement rate for Tweets posted by the Society of Behavioral Medicine Twitter account (@BehavioralMed) was obtained directly from Twitter. The engagement rate for posts from other accounts hosting comparison Twitter Chats was obtained via Popsters, a commercial social media analytics tool [16]. Engagement rates were examined for posts by the chat host presenting the discussion questions for each Twitter Chat. These posts were identified by manually reviewing tweets from the host accounts on the date of each chat.

Of the four questions posted during the cardio-oncology Twitter Chat, question #1 had the highest engagement rate (66 engagements/3935 impressions = 1.7%). The next highest engagement rate was for question #3 (53 engagements/3611 impressions = 1.5%), followed by questions #2 (41 engagements/3278 impressions = 1.3%) and #4 (22 engagements/2802 impressions = 0.8%). For reference, the median Twitter engagement rate across industries is 0.045% [17]. Specific to medically-related Twitter Chats, the abovementioned #HFChat2020 had 10 discussion questions with engagement rates ranging from 0.03–0.2%. Engagement rates for the discussion questions posted in the three prior SBM Twitter Chats were 0.2–0.7% (February 18, 2020), 0.4–0.7% (December 9, 2019), and 0.1–1.5% (December 6, 2019). The higher than average engagement rate observed in the cardio-oncology chat suggests that the chat questions were highly relevant to the target audience.

Several other factors may have contributed to the high engagement rate for the cardio-oncology chat. First, in reviewing the top tweets from the cardio-oncology Twitter Chat, tweets generally fell into four categories: 1) knowledge impartation (K), 2) advocacy awareness (A), 3) interdisciplinary collaboration (I), and 4) learning impact (L), which we have termed “KAIL”. Examples of each category are provided in Fig. 1. We believe that these four components contributed to the high engagement rate in this Twitter Chat. Second, our guests noted that they were very intentional in composing their tweets, prepared the content in advance, and provided eye-catching graphics and references. Third, many popular posts from the cardio-oncology Twitter Chat included links to academic publications providing research results related to cardio-oncology. Given the positive response to these types of informational posts during the cardio-oncology Twitter Chat, we believe that Twitter Chats can play an important role in spreading awareness about cardio-oncology.

The emerging role of social media in general – and Twitter in particular – in the dissemination of health information has been recognized for some time [18, 19]. Twitter in particular has become a forum for communication among health care clinicians, scientists, and patients. Additionally, social media users frequently turn to these platforms for health information [20]. Given the potential benefits for both patients and professional communities, health care professionals and academics are increasingly encouraged to develop and maintain a social media presence [21–23].

However, less is known about the most effective ways to disseminate high-quality health information through social media. In one qualitative study of 17 physicians, participants frequently described uncertainty about strategies for social media use [24]. Furthermore, participants described using social media much like traditional media, as a one-way communication platform, rather than as an interactive forum.

We submit that Twitter Chats help overcome many of the pitfalls of social media use for health care professionals. Specifically, Twitter Chats have three primary advantages. First, Twitter Chats are inherently structured to facilitate engagement and two-way communication. Second, the simultaneous use of a designated hashtag by all participants during the Twitter Chat helps facilitate real-time conversation about a specific topic. Finally, by bringing together a diverse group of guests and participants – each with their own followers – Twitter Chats can expand the reach of posts far beyond the audience of a single user.

In sum, Twitter Chats inexpensively utilize innovative technology to provide education and foster community. Long-term studies are needed to understand whether Twitter Chats can change knowledge and behavior related to cardio-oncology. The impact of Twitter Chats on knowledge dissemination could be operationalized as an increase in altmetrics (metrics for scholarly publications and activities derived from the social web [25, 26]), online access/page views or downloads [27], or citation counts [26, 28, 29] for scientific research articles mentioned during the chat (versus similar articles not mentioned during the chat). The impact of social media mentions on these outcomes has been previously examined [26–29], but not in the context of a Twitter Chat which has unique advantages over traditional asynchronous use of social media. Given the timeframe of peer review and publication of scholarly journal articles [30], long-term follow-up (12–30 months) may be necessary to see an impact of Twitter Chats on citation counts. Alternatively, a deidentified survey may be used to collect follow-up data from chat participants regarding changes in knowledge of cardio-oncology, as has been done previously for other topic areas [31]. Finally, future research might extend the data presented here by using social network analysis to examine “return on engagement”, a metric developed to analyze whether Twitter Chats facilitate two-way communication [32]. Studies such as these would provide additional support for the efficacy of Twitter Chats as a mechanism for education, outreach, and advocacy.

## Data Availability

Data presented are publicly available on the websites Twitter.com and Symplur.com.

## Abbreviations

MD: Doctor of Medicine
RD: Registered dietitian
RSW: Registered social worker
KAIL: Acronym for domains identified in the cardio-oncology Twitter Chat, stands for “knowledge impartation (K), advocacy awareness (A), interdisciplinary collaboration (I), and learning impact (L)”

## Footnotes

1 A “hashtag” is a phrase introduced by the number sign, pound sign, or hash symbol (#) that acts as a keyword for a post. Hashtags are searchable and allow other users to easily find messages with a specific theme or content.

2 Consistent with the social media policies of the Society of Behavioral Medicine, the impressions and engagement statistics presented here were obtained *without* the use of paid amplification tools (e.g., “promoted” Tweets).

## References

[1] Brown S-A (2020). Preventive cardio-oncology: the time has come. Front Cardiovasc Med.

[2] Bellinger AM, Arteaga CL, Force T, Humphreys BD, Demetri GD, Druker BJ (2015). Cardio-oncology: how new targeted cancer therapies and precision medicine can inform cardiovascular discovery. Circulation.

[3] Yeh ET, Bickford CL (2009). Cardiovascular complications of cancer therapy: incidence, pathogenesis, diagnosis, and management. J Am Coll Cardiol.

[4] Daher IN, Daigle TR, Bhatia N, Durand J-B (2012). The prevention of cardiovascular disease in cancer survivors. Tex Heart Inst J.

[5] Fradley MG, Brown AC, Shields B, Viganego F, Damrongwatanasuk R, Patel AA, et al. Developing a comprehensive cardio-oncology program at a cancer institute: the Moffitt Cancer Center experience. Oncol Rev. 2017;11(2):340. 10.4081/oncol.2017.340 PMC5523022 28781723

[6] Parent S, Pituskin E, Paterson DI (2016). The cardio-oncology program: a multidisciplinary approach to the care of cancer patients with cardiovascular disease. Can J Cardiol.

[7] Syed IA, Klassen AF, Barr R, Wang R, Dix D, Nelson M (2016). Factors associated with childhood cancer survivors’ knowledge about their diagnosis, treatment, and risk for late effects. J Cancer Surviv.

[8] Ruud E, Kanellopoulos A, Zeller B, Widing E, Tjønnfjord G, Fosså S (2012). Patient knowledge of late effects of acute lymphoblastic leukaemia. Tidsskrift for den Norske laegeforening: tidsskrift for praktisk medicin, ny raekke.

[9] Geramita EM, Parker IR, Brufsky JW, Diergaarde B, van Londen G. Primary care providers’ knowledge, attitudes, beliefs, and practices regarding their preparedness to provide Cancer survivorship care. J Cancer Educ. 2019. 10.1007/s13187-019-01585-4. 10.1007/s13187-019-01585-4 PMC8266289 31388974

[10] Peng J, Rushton M, Johnson C, Brezden-Masley C, Sulpher J, Chiu MG (2019). An international survey of healthcare providers’ knowledge of cardiac complications of cancer treatments. Cardio-Oncology.

[11] Mandrola J, Futyma P (2020). The role of social media in cardiology. Trends Cardiovasc Med.

[12] "Demographics of Social Media Users and Adoption in the United States". Washington, D.C.: Pew Research Center; 2019. https://www.pewresearch.org/internet/fact-sheet/social-media/.

[13] Brown S-A, Daly RP, Duma N, Yang EH, Pemmaraju N, Parwani P (2020). Leveraging social media for cardio-oncology. Curr Treat Options in Oncol.

[14] Brown S-A, Rhee J-W, Guha A, Rao VU, Innovation in Precision Cardio-Oncology During the Coronavirus Pandemic and Into a Post-pandemic World. Front Cardiovasc Med. 2020;7. 10.3389/fcvm.2020.00145 PMC7456950 32923460

[15] Symplur. Healthcare Hashtag Project 2020 [Available from: https://www.symplur.com/healthcare-hashtags/.

[16] Popsters. Popsters: Social Media Content Analytics Tool 2020 [Available from: https://popsters.com/.

[17] RivalIQ. 2020 Social Media Industry Benchmark Report 2020 [updated March 4, 2020. Available from: https://www.rivaliq.com/blog/social-media-industry-benchmark-report/.

[18] Walsh MN (2018). Social media and cardiology. J Am Coll Cardiol.

[19] Sedrak MS, Cohen RB, Merchant RM, Schapira MM (2016). Cancer communication in the social media age. JAMA Oncol.

[20] Househ M, Borycki E, Kushniruk A (2014). Empowering patients through social media: the benefits and challenges. Health Inform J.

[21] Parwani P, Choi AD, Lopez-Mattei J, Raza S, Chen T, Narang A (2019). Understanding social media: opportunities for cardiovascular medicine. J Am Coll Cardiol.

[22] Cabrera D, Vartabedian BS, Spinner RJ, Jordan BL, Aase LA, Timimi FK (2017). More than likes and tweets: creating social media portfolios for academic promotion and tenure. J Grad Med Educ.

[23] Chan TM, Stukus D, Leppink J, Duque L, Bigham BL, Mehta N (2018). Social media and the 21st-century scholar: how you can harness social media to amplify your career. J Am Coll Radiol.

[24] Campbell L, Evans Y, Pumper M, Moreno MA (2016). Social media use by physicians: a qualitative study of the new frontier of medicine. BMC Med Inform Decis Mak.

[25] Priem J, Piwowar HA, Hemminger BM. Altmetrics in the wild: Using social media to explore scholarly impact. arXiv:1203.4745 [Preprint]. 2012. Available from: http://arxiv.org/html/1203.4745v1.

[26] Xia F, Su X, Wang W, Zhang C, Ning Z, Lee I (2016). Bibliographic analysis of nature based on twitter and Facebook altmetrics data. PLoS One.

[27] Widmer RJ, Mandrekar J, Ward A, Aase LA, Lanier WL, Timimi FK (2019). Effect of promotion via social media on access of articles in an academic medical journal: a randomized controlled trial. Acad Med.

[28] Eysenbach G (2011). Can tweets predict citations? Metrics of social impact based on twitter and correlation with traditional metrics of scientific impact. J Med Internet Res.

[29] Finch T, O'Hanlon N, Dudley SP (2017). Tweeting birds: online mentions predict future citations in ornithology. R Soc Open Sci.

[30] Björk B-C, Solomon D (2013). The publishing delay in scholarly peer-reviewed journals. J Informetrics.

[31] Attai DJ, Cowher MS, Al-Hamadani M, Schoger JM, Staley AC, Landercasper J (2015). Twitter social media is an effective tool for breast cancer patient education and support: patient-reported outcomes by survey. J Med Internet Res.

[32] Rabarison KM, Croston MA, Englar NK, Bish CL, Flynn SM, Johnson CC (2017). Measuring audience engagement for public health twitter chats: insights from# LiveFitNOLA. JMIR Public Health surveill.

